# Effects of dexmedetomidine on TNF-α and interleukin-2 in serum of rats with severe craniocerebral injury

**DOI:** 10.1186/s12871-017-0410-7

**Published:** 2017-09-20

**Authors:** Wan-Wei Jiang, Qing-Hui Wang, Ya-Jing Liao, Pai Peng, Min Xu, Li-Xin Yin

**Affiliations:** Department of Anesthesiology II, Affiliated Zhongshan Hospital of Dalian University, No. 6 of Jiefang Street, Zhongshan District, Dalian, 116001 China

**Keywords:** Dexmedetomidine, Severe craniocerebral injury, TNF-α, Il-2

## Abstract

**Background:**

Dexmedetomidine is a highly selective adrenergic receptor agonist, which has a dose-dependent sedative hypnotic effect. Furthermore, it also has pharmacological properties, and the ability to inhibit sympathetic activity and improve cardiovascular stability during an operation. However, its protective effect on patients with severe craniocerebral injury in the perioperative period remains unclear.

**Method:**

Eighty adult male SD rats were used and divided into two groups (*n* = 40, each group): dexmedetomidine injury group (experimental group), and sodium chloride injury group (control group). Models of severe craniocerebral injury were established in these two groups using the modified Feeney’s free-fall method. As soon as the establishment of models was succeed, rat in the experimental group received 1 μg of dexmedetomidine (0.1 ml), while each rat in the control group was given 0.1 ml of 0.9% sodium chloride. Blood was sampled from an incision at the femoral vein to detect TNF-α and IL-2 levels at 1, 12, 24,36,48 and 72 h after establishing the model in the two groups.

**Results:**

After severe craniocerebral injury, TNF-α levels of rats were lower in every stage and at different degrees in the experimental group than in the control group (*P* < 0.05), while IL-2 levels were lower in the experimental group to different extents (*P* < 0.05).

**Conclusion:**

Dexmedetomidine protects the brain of rats with severe craniocerebral injury by reducing the release of inflammatory mediators.

## Background

In recent years, the number of studies on dexmedetomidine has increased. Increasing studies [1–5] have revealed that dexmedetomidine has organ-protecting and anti-inflammatory effects [6]. Furthermore, a previous study [7] has demonstrated that this drug could prevent local ischemic nerve injury in animals with transient brain ischemia. Although the exact mechanism remains unknown, researchers have speculated that this might be related to the decrease in catecholamine concentration outside the brain cells, the regulation of apoptosis, and the decrease in excitatory neurotransmitter glutamate. Therefore, this mechanism requires further studies. Traumatic brain injury (TBI) is a common clinical emergency. In particular, severe craniocerebral injury has high mortality and disability rates, which has been the focus of clinical attention [8]. One of the main causes of the poor clinical prognosis of this injury is secondary brain injury (SBI), which follows primary brain injury. The main manifestations of SBI include the following: destruction of the blood brain barrier, secondary ischemic and hypoxic injury, and cerebral edema. At present, it has been considered that secondary inflammatory response after craniocerebral injury is an important cause of SBI [9–11]. In recent years, studies on cytokines have confirmed that inflammatory cytokines are involved in secondary injury after TBI.

As important inflammatory response factors, tumor necrosis factor-α (TNF-α) and interleukin-2 (IL-2) play important roles in the pathological process of craniocerebral injury. TNF-α is the earliest and most important inflammatory mediator in inflammatory response, which activates neutrophils and lymphocytes, increases the permeability of vascular endothelial cells, regulates the metabolism of other tissues, and promotes the synthesis and release of other cytokines. IL-2 is an important immune regulatory factor produced by helper T cells, which promotes the proliferation of T cells, induces the growth of LAK cells, promotes B cells to secrete antibodies, promotes the killing effect of Tc cells, and enhances the activity of natural killing (NK) cells; and in particular, promotes the secretion of interferons [12]. Therefore, IL-2 is a central link in the regulation of immune cell proliferation and response; and its level can reflect the activity of cellular immunity. When the body’s cellular immune function is suppressed, the lymphocyte transformation level and induced production of IL-2 are significantly decreased [13–15].

Dexmedetomidine is a highly selective adrenergic receptor agonist, which has a dose-dependent sedative hypnotic effect. Furthermore, it also has pharmacological properties such as analgesia, and the ability to inhibit sympathetic activity and improve cardiovascular stability during an operation. However, its protective effect on patients with severe craniocerebral injury in the perioperative period remains unclear. This study aims to investigate its protective effect on the brain of rats with severe craniocerebral injury.

## Methods

### Experimental animals

Eighty healthy 3–4 month-old Sprague Dawley (SD) rats were selected for this study. Rats were of laboratory animal grade 1; and the body weight of these rats ranged between 250 and 300 g. Rats were randomly divided into two groups (*n* = 40, each group): dexmedetomidine group (experimental group) and sodium chloride group (control group).

### Main experimental materials and reagents

Small animal striking devices were used in this study. The enzyme-linked immunosorbent assay (ELISA) kit was purchased from Beijing Huatai Rongxin Biotechnology Co., Ltd., which was produced in Calabasas, California, USA. Operation procedures were referred to the specifications of the kit.

### Establishment of the severe craniocerebral injury model

Models were established using the Feeney’s free-fall epidural impact method [16]. Before inducing the injury, animals were anesthetized by intraperitoneally injecting 2% chloralic hydras (45 mg/kg). After success of the anesthesia, animals were placed in the prone position with heads fixed on the operating table. Hair on top of the head was removed and an incision on the scalp in the middle of the sagittal section was performed after sterilization. Then, the periosteum was stripped to expose the left parietal bone. A small hole was made using a dental drill at the site 2 mm before the lambdoid suture and 2 mm on the left from the midline. Next, the small hole was expanded to a 5-mm diameter bone window, and the integrity of the dura was maintained during this process. A pad (with a diameter of approximately 4 mm) was placed on the dura, a 40-g hammer was set free from a height of 30 cm, fell along the cannula, and impacted on the pad; causing a regional cerebral contusion and laceration on the parietal lobe. The bone window was sealed with bone wax, and the scalp was sutured. Animal mortality rate was 25% for this process.

As soon as the establishment of models was succeed, Rats in the experimental group received 1 μg of dexmedetomidine (0.1 ml), while rats in the control group were given 0.1 ml of 0.9% sodium chloride.

### Detection methods

After the animal models were successfully established, at time points of 1, 12, 24, 36, 48 and 72 h, the tails were cut for blood sampling. Then, serum was obtained by isolation, and preserved at −20 °C. Changes in TNF-α and IL-2 were detected by ELISA after all samples were collected.

### Statistical methods

All data were analyzed using statistical software SPSS 10.0. Measurement data were expressed as mean ± standard deviation (x ± SD). Data from groups with different injury levels were compared using analysis of variance. When variance between groups was not homogeneous, data were compared using non-parametric Kruskal-Wallis rank sum test. Data of the same index acquired at different time points were compared using repeated measures analysis of variance. Further multiple comparisons were performed using repeated measures analysis of variance and Dunnett’s *t*-test.

## Results

### General situations

After brain impact, most of the rats experienced apnoea, twitch and gatism. Animals with moderate and severe brain injury mostly presented with continuous twitch and extreme rigidity, had the inability to turn over, and had instable standing or walking ability; which was more significant on the left leg. One rat died in the experimental group after 48 h, and was replaced with a new rat.

### Dynamic changes of serum TNF-α and IL-2 levels

**Fig. 1:**
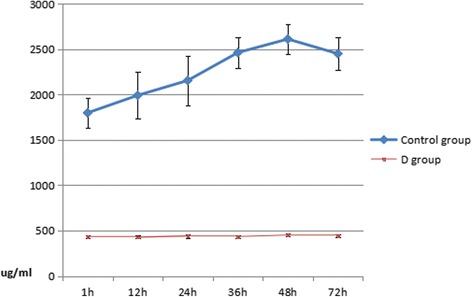
Dynamic changes of serum TNF-α at different times

**Fig. 2:**
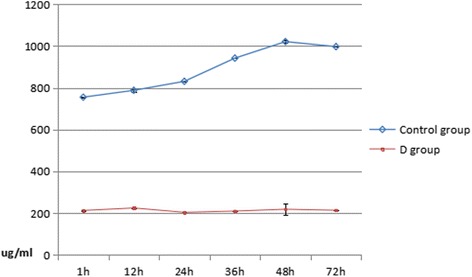
Dynamic changes of serum IL-2 at different times

Serum TNF-α and IL-2 levels rapidly increased in rats in the two groups Figs. 1 and 2. These levels were significantly lower in the experimental group than in the control group (*P* < 0.01). The concentration of the IL-2 in the two groups both increased one hour after the injury. But the IL-2 level in the experimental group significantly decreased at each time point compared to the control group, and the difference was statistically significant (*P* < 0.05). The concentrations of TNF-α in the two groups both increased one hour after the injury. The TNF-α level in the experimental group also significantly decreased at each time point compared to the control group, and the difference was statistically significant (*P* < 0.05). Details are shown in Tables 1 and 2.

**Table 1:** Dynamic changes of serum TNF-α at different times(μg/ml, mean ± SD)

Groups	n	1 h	12 h	24 h	36 h	48 h	72 h
Control group	40	1798. ± 163.44	1991 ± 259.00	2155 ± 274.82	2462 ± 173.63	2611 ± 163.40	2450 ± 182.53
D group	40	435 ± 15.38	428 ± 15.38	441 ± 20.14	435 ± 14.55	451 ± 13.88	446 ± 14.33

**Table 2:** Dynamic changes of serum IL-2 at different times(μg/ml, mean ± SD)

Groups	n	1 h	12 h	24 h	36 h	48 h	72 h
Control group	40	755 ± 2.33	788 ± 6.55	831 ± 0.22	942 ± 0.32	1021 ± 6.17	997 ± 0.17
D group	40	213 ± 3.10	225 ± 0.38	204 ± 0.43	210 ± 0.63	219 ± 28.47	214 ± 0.83

## Discussion

Present studies have suggested that nerve injury in early severe craniocerebral injury is an excessive inflammatory response. Inflammatory response is a double-edged sword in the body, which is designed to remove alien and necrotic cells. However, excessive inflammatory response causes cerebral ischemia-reperfusion injury and aggravates brain edema, nerve cell necrosis and apoptosis; which is an important factor for secondary brain damage after severe craniocerebral injury [17–20].

The central nervous system (CNS) of rats with severe craniocerebral injury can produce a large amount of IL-2 and TNF-α, as well as other inflammatory cytokines [21–23]. As initiation factors, these inflammatory cytokines cause secondary inflammatory responses after craniocerebral injury, and result in secondary brain damage. IL-2 and TNF-α plays a core role in responses induced by severe craniocerebral injury [24].

IL-2 plays an important role in inflammatory response. In the early stage of craniocerebral injury, serum IL-2 level significantly increases. This is related to the severity of damage at that period, which reaches the first peak at 36 h after injury and reaches the highest peak at 48 h after injury. Then, this level decreases to a certain extent at 72 h after injury, but the level is significantly lower than the control group. There was no obvious acute infection during the early stage of closed craniocerebral injury, and the production of IL-2 may be mainly caused by the mechanism of traumatic stress reaction. After injury, inflammatory response, the regulation of immune function and the secretion of a variety of cytokines would reach a new balanced status in a short period of time, instead of a persistent disturbance status. Therefore, plasma IL-2 levels would rapidly increase and subsequently rapidly decrease after a short period of time.

Some previous studies have revealed that dexmedetomidine could significantly inhibit the production of TNF-α and IL-6, and improve the prognosis of inflammation in rats [25–29]. Most researchers have considered that inflammation plays an important role in the development of brain damage after craniocerebral injury [30, 31], and TNF-α could be used as an independent evaluation index in this process. It is possible that TNF-α directly affects the condition and prognosis of craniocerebral injury through the activation of its autoreceptor and complement system. This would induce the expression of endothelial cells, adhesion molecules and monocyte tissue factors, as well as the activation of monocytes, to release inflammatory cytokines such as IL-2 and TNF-α [32–34]; and directly cause inflammation. TNF-α level is closely related to the repair of tissues after injury. This experiment revealed that TNF-α levels significantly increased at 24 h after severe craniocerebral injury, which reached the first peak at 36 h after injury. This levels reached the second peak at 48 h after injury, and continued to increase at 72 h after injury. In the experimental group the TNF-α level was much lower than the control group.

The results of this experiment revealed that the TNF-αand IL-2 levels both decreased in the experimental group compared to the control group. Since TNF-α and IL-2 play important roles in the secondary brain damage after severe craniocerebral injury, dexmedetomidine can protect the brain in the rats with craniocerebral injury by decreasing the levels of TNF-α and IL-2.

## Conclusion

In summary, dexmedetomidine plays a role in brain protection by reducing the release of inflammatory mediators during severe craniocerebral injury.

## Abbreviations

ELISA: enzyme-linked immunosorbent assay
IL-2: interleukin-2
NSS: neurological severity scores
SBI: secondary brain injury
TBI: traumatic brain injury
TNF-α: tumor necrosis factor-α.
